# PtdIns(4,5)P2 and PtdIns(3,4,5)P3 dynamics during focal adhesions assembly and disassembly in a cancer cell line

**DOI:** 10.3906/biy-2004-108

**Published:** 2020-12-14

**Authors:** Dhurgham ALFAHAD, Salem ALHARETHI, Bandar ALHARBI, Khatab MAWLOOD, Philip DASH

**Affiliations:** 1 Department of Pathological Analysis, College of Science, Thi-Qar University, Thi-Qar Iraq; 2 Department of Biological Science, College of Arts and Science, Najran University, Najran Saudi Arabia; 3 Department of Clinical Laboratory Sciences, College of Applied Medical Science, University of Hail, Hail Saudi Arabia; 4 Department of Biology, College of Science, Soran University, Irbil Iraq; 5 Department of Biomedical Sciences, School of Biological Sciences, University of Reading, Reading United Kingdom

**Keywords:** Focal adhesions turnover, PtdIns(4,5)P2, PtdIns(3,4,5)P3

## Abstract

Focal adhesions (FAs) are large assemblies of proteins that mediate intracellular signals between the cytoskeleton and the extracellular matrix (ECM). The turnover of FA proteins plays a critical regulatory role in cancer cell migration. Plasma membrane lipids locally generated or broken down by different inositide kinases and phosphatase enzymes to activate and recruit proteins to specific regions in the plasma membrane. Presently, little attention has been given to the use of phosphatidylinositol 4,5-bisphosphate (PtdIns(4,5)P2) and Phosphatidylinositol 3,4,5-trisphosphate (PtdIns(3,4,5)P3) fluorescent biosensors in order to determine the spatiotemporal organisation of PtdIns(4,5)P2 and PtdIns(3,4,5)P3 within and around or during assembly and disassembly of FAs. In this study, specific biosensors were used to detect PtdIns(4,5)P2, PtdIns(3,4,5)P3, and FAs proteins conjugated to RFP/GFP in order to monitor changes of PtdIns(4,5)P2 and PtdIns(3,4,5)P3 levels within FAs. We demonstrated that the localisation of PtdIns(4,5)P2 and PtdIns(3,4,5)P3 were moderately correlated with that of FA proteins. Furthermore, we demonstrate that local levels of PtdIns(4,5)P2 increased within FA assembly and declined within FA disassembly. However, PtdIns(3,4,5)P3 levels remained constant within FAs assembly and disassembly. In conclusion, this study shows that PtdIns(4,5)P2 and PtdIns(3,4,5)P3 localised in FAs may be regulated differently during FA assembly and disassembly.

## 1. Introduction

Focal adhesions (FAs) have been shown to be involved in many aspects of cell physiology, such as signal transduction (Morimatsu et al., 2015), the anchoring of cells to ECM, and the cell’s response to mechanical and physical forces (Geiger et al., 2009; Hoffman et al., 2011). PtdIns(4,5)P2 and PtdIns(3,4,5)P3 are lipids measuring 64.5 ± 28 nm and 125.6 ± 22 nm, respectively; both can be found in distinct and well-restricted regions of pheochromocytorna (PC12) cell membranes (Wang & Richards, 2012). Many signalling pathways have been shown to be regulated by PtdIns(4,5) P2 and PtdIns(3,4,5)P3 through the recruitment of kinases into these regions (Wang & Richards, 2012). For example, Phosphatidylinositol 4-phosphate 5-kinase 1 (PIPK1) and phosphoinositide 3-kinase (PI3K) are known to regulate Fas by synthesising PtdIns(4,5)P2 and PtdIns(3,4,5)P3 (Legate et al., 2011; Wu et al., 2011; Nader et al., 2016). FAs play a crucial role in cell migration and undergo dynamic changes that involve many temporal stages (Fogh et al., 2014). In the course of such temporal stages, many kinases and other enzymes, such as phosphatidylinositol 4-phosphate 5-kinase γ(PIP5Kγ90), PI3K, and phospholipase C (PLC), are activated and recruited by FA proteins into FA sites. In turn, once kinases such as PIP5Kγ90 are activated by FA proteins (e.g., talin), this subsequently leads to the production of PtdIns(4,5)P2 pools in the plasma membrane (Legate et al., 2011; Zhaofei Wu et al., 2011; Izard & Brown, 2016). These PtdIns(4,5) P2 pools play an important role in the formation of FAs (Garvalov et al., 2003; Carisey & Ballestrem, 2011). At later stages of FA formation, FA proteins activate each other to form a bridge between the actin cytoskeleton and integrins of which the strength can be increased by pools of PtdIns(4,5)P2 (Izard & Brown, 2016).

**Figure 1:**
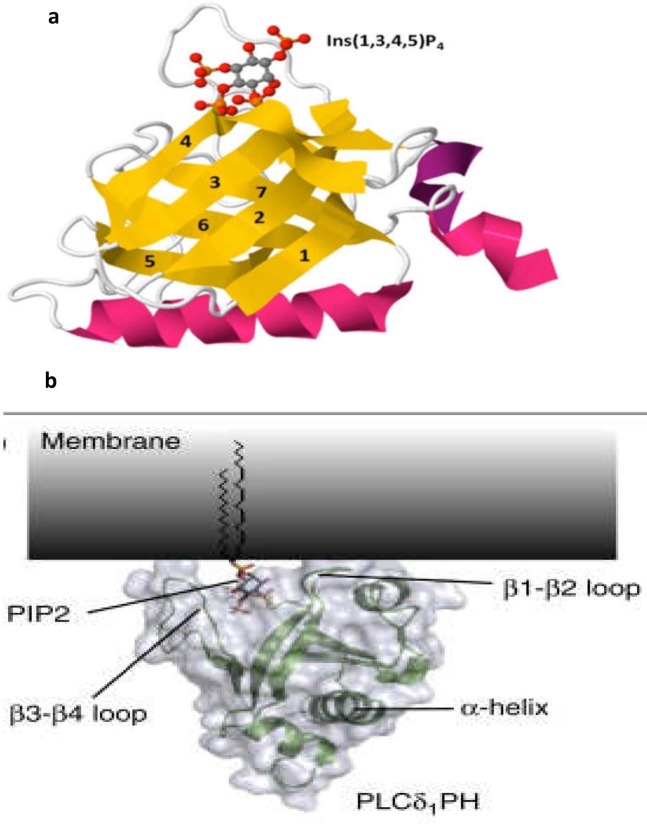
Structure of two ph domains in interaction with ptdins. The PH domain consists of an α-helix (pink colour) and 7 β-strands (yellow colour), forming a β-barrel. PI binds to the PH domain through the loops of the β-barrel. (a) Btk-PH-Ins(1,3,4,5)P4 interaction; (b) PLC𝛅-PH PtdIns(4,5)P2 interaction (Bunney and Katan, 2011; Wang et al., 2015).

Pleckstrin Homology (PH) domains consist of around 120 amino acids and have a seven-strand β-barrel which forms 2 antiparallel β-sheets and a C-terminal α-helix (Figure 1). The β1, β2, β3, and β4 loops form specific sites where the inositol ring of phosphoinositide binds (Wang et al., 2015). Approximately 10% of all PH domains possess high affinity and selectivity for phosphoinositides (Lemmon, 2007). PH domains of Phospholipase C delta (PLCδ) and Bruton’s tyrosine kinase (Btk) have high affinity and selectivity for PtdIns(4,5)P2 and PtdIns(3,4,5)P3, respectively. This is due to neighbouring phosphates in their inositol ring. Other types of phosphoinositides, such as phosphatidylinositol 3-phosphate(PtdIns3P), phosphatidylinositol 5-phosphate (PtdIns5P), and phosphatidylinositol 3,5-bisphosphate (PtdIns(3,5)P2) bind to domains, such as FYVE domains, PHD finger, and PX (Lemmon, 2007). This diversity of phosphoinositide selectivity on distinct types of PH domains is important to recruit a variety of kinases to the plasma membrane, where they will carry out their function and interact with other signalling pathways (Lemmon, 2007). Some of these kinases phosphorylate the inositol ring to generate a new phosphoinositide, PI3K phosphorylate PtdIns(4,5) P2, into PtdIns(3,4,5)P3. New second messengers such as inositol trisphosphate (IP3) and diacylglycerol (DAG) are produced as a result of PLC activity on PtdIns(4,5)P2. Other kinases, such as PTENde, phosphorylates different sites on the inositol ring of PtdIns(3,4,5)P3 to produce PtdIns(4,5)P2(Lemmon, 2007, Wang et al., 2015). While the role of FA proteins PtdIns(4,5)P2 and PtdIns(3,4,5) P3 have previously been investigated, little attention has been given to quantify the local changes of PtdIns(4,5)P2 or PtdIns(3,4,5)P3 levels within and around FAs during their assembly and disassembly in MDA-MB-231 cells given the metastatic and high motility proprieties of the latter (Cailleau et al., 1974). Thus, the aim of this study is to investigate the spatial distribution of PtdIns(4,5)P2 and PtdIns(3,4,5)P3 in relation to the localisation of FA proteins such as zyxin and paxillin, followed by a quantification of the levels of PtdIns(4,5)P2 and PtdIns(3,4,5)P3 during the assembly and disassembly of FAs in MDA-MB-231 cells.

## 2. Materials and methods

### 2.1. Cell lines and cell culture

All experiments were performed using MDA-MB-231 (ATCC HTB­26) cells purchased from the cell bank of the American Type Culture Collection cell bank (Manassas, USA). These MDA-MB-231 cells were grown in high glucose Dulbecco’s Modified Eagle’s Medium (DMEM, Gibco), supplemented with L-glutamine, 10% (v/v) foetal bovine serum (FBS, Gibco), and 1% v/v penicillin/streptomycin at 37 °C in an atmosphere containing 95% air, 5% CO2, and ~90% humidity. Regular testing for mycoplasma was performed on these cells with the aid of an EZ-PCR Mycoplasma Test kit (Cat No. K1-0210, EZ-PCR Mycoplasma Test Kit, 20 assays, Geneflow, UK), following the manufacturer’s instruction.

### 2.2. Construction of fluorescent biosensors

To generate the Btk-PH-mCherry biosensor, both Btk-PH-GFP (Addgene; Plasmid #51463) and mCherry-N1 (TAKARA; #632523) were digested with *BamH1* and *EcoR1* restriction enzymes; the resulting Btk-PH fragment was ligated to the mCherry-N1 construct (TAKARA #632523) at the restriction sites mentioned above. A fragment of PLC;;1-PH was amplified by polymerase chain reaction (PCR) using PLC;;1-PH-GFP (Addgene; Plasmid #21179) as template and 2 primers: 5’-AGCTAGATCTTTACTGGATGTTGAGCTCCT-3’(forward) and 5’-AAGTCCCGGCCCGAGTCCATGGATCCAGCT-3’(reverse). The resulting PCR product was subsequently digested with *Bgl11* and *EcoR1* , then cloned into the *Bgl11* and *EcoR1* sites of the mCherry-C1 construct (Addgene; #632524) in order to generate a construct encoding the PLC;;1-PH-mCherry biosensor.

### 2.3. Transfection of MDA-MB-231 cells and live cell imaging

A day prior to transfection, 2 mL of 1 × 105 MDA-MB-231 cells/mL suspended in supplemented DMEM were seeded into culture dishes upon which were placed glass cover slips that were coated with either ECM collagen (BD Bioscience) or fibronectin (BD Bioscience) or gelatine (Sigma Aldrich). Sterile glass cover slips were treated either with 0.2% w/v gelatine (diluted in PBS) for 20 min, with 10-µɡ/mL fibronectin (diluted in PBS) for 1 h, or with 2-mg/mL rat tail type 1 nonpepsinised collagen (diluted in DMEM; pH adjusted to 7.0 with NaOH) for approximately 15 min. MDA-MB-231 cells were transfected with the aid of Polyethylenimine (PEI) (Sigma Aldrich). Approximately 100 µL of serum-free DMEM was used to dilute 3-µɡ plasmid DNA consisting of either zyxin-RFP (Addgene; plasmid #26720) or paxillin-RFP (a construct designed in Prof. Phil Dash’s laboratory, University of Reading) and GFP-C1-PLCδ1-PH or Btk-PH-GFP (Addgene; Plasmid #51463) at a w/w ratio of 1:1. Following the addition of 6 µL of PEI to the DNA diluted in DMEM, the reaction was left to incubate for 15 min. The PEI/DNA mixture was then added to the cells and was left to incubate for 24 h. Live cells were visualised using a confocal microscope (Nikon Eclipse Ti Laser-scanner), coupled with a warm chamber (37 °C) perfused with 5% CO2. Fluorescence of GFP and RFP (or mCherry) was detected at 488 nm/510 nm and 568 nm/590 nm (excitation/emission), respectively. The laser intensity and exposure time were adjusted to reduce the signal-to-noise ratio of the fluorescence.

### 2.4. Immunofluorescence staining

Following transfection, MDA-MB-231 cells cultured on glass coverslips with different cells surfaces were washed with PBS supplemented with Ca2+ and Mg2+, then fixed for 20 min with 4% PFA (diluted in PBS) at room temperature. The cells were subsequently washed 3 times with PBS (10 min each), followed by a 10 min permeabilization with 0.5% Triton-X 100 (diluted in PBS). After 30 min of blocking-10% goat serum (diluted in 0.5% Triton-X 100), the cells were incubated for 1 h with either an antizyxin antibody (Abcam; ab50391) (1:100) or antipaxillin antibody (Abcam; ab23510) (1:100), both diluted in 2% goat serum. Excess of primary antibodies was removed by 3 sequential washes with PBS (10 min each); thereafter, the cells were incubated for 1 h with an Alexa Fluor 488 conjugated to a rabbit anti-IgG antibody (Cell Signalling; 4412; 1:100). After the removal of unbound secondary antibodies with 3 sequential washes (PBS; 10 min each time), the nuclei were stained, and the cells were mounted onto glass slides with the Fluoroshield mounting medium containing DAPI. The cells were visualised by confocal microscopy (Nikon Eclipse Ti Laser-scanner). The DAPI was detected at 350 nm/470 nm (excitation/emission) while the Alexa Fluor 488 was detected at 488 nm/510 nm (excitation/emission).

### 2.5. Colocalisation and local lipid quantification within FA analysis

**Figure 2:**
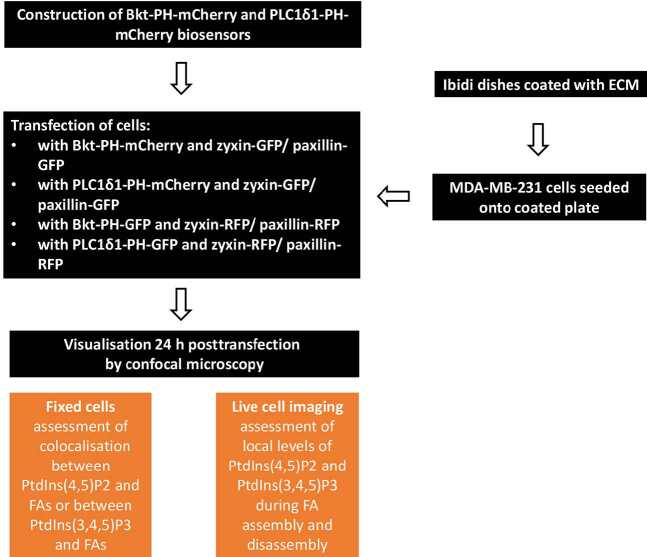
Flow diagram depicting the experimental procedures. This diagram shows the design of PtdIns(4,5)P2 and PtdIns(3,4,5)P3 biosensors, which were in turn used together with FAs (zyxin/ paxillin)-GFP or -RFP fusion genes to transfect MDA-MB-231 cells cultured upon different cell surfaces. Fixed and live cells were visualised by confocal microscopy to assess the colocalisation between FA proteins and PtdIns(4,5)P2/PtdIns(3,4,5)P3, as well as to quantify the local levels of both PtdIns(4,5)P2 and PtdIns(3,4,5)P3 during FA assembly and disassembly.

Colocalisation analyses between PtdIns(4,5)P2 and FAs or PtdIns(3,4,5)P3 and Fas were performed with the aid of Image J software on images acquired from fixed and live cells. All images were adjusted to 8-bit colour graphics and the individual image was separated into 2 channels: one containing fluorescence from RFP-FA and the other containing fluorescence from either GFP-PtdIns(4,5)P2 or GFP-PtdIns(3,4,5)P3. Improved signal-to-noise was obtained by processing each image through background fluorescence subtraction. Individual FA was then analysed after magnification and the colocalisation coefficient was determined using the Spearman’s rank correlation coefficient, which was measured between PtdIns(4,5)P2/PtdIns(3,4,5)P3, and FA. Quantifications of PtdIns(4,5)P2, PtdIns(3,4,5)P3, and FA were performed within a region of interest (ROI) selected in the surrounding vicinity of individual FA using Image J. The ROI was investigated with regard to RFP fluorescence–in relation to FA and GFP fluorescence–in relation to either PtdIns(4,5)P2 or PtdIns(3,4,5)P3. The intensity of fluorescence of PtdIns(4,5)P2 or PtdIns(3,4,5)P3 was measured throughout the lifetime of FAs. All experimental methods are summarised as a schematic figure (Figure 2).

### 2.6. Statistical analysis

All statistical analyses were performed with GraphPad Prism 5 software (GraphPad Software, San Diego, CA). Upon significant difference determined by one-way ANOVA(P < 0.05), a posthoc analysis was performed using either Tukey’s or Dunn’s multiple comparisons test. The P-values <0.05 were considered statistically significant. All results comprise data from triplicates of at least 3 independent experiments (N = 3).

## 3. Results

### 2.1. Spatial distribution of PtdIns(4,5)P2 and PtdIns(3,4,5)P3 within FAs

**Figure 3:**
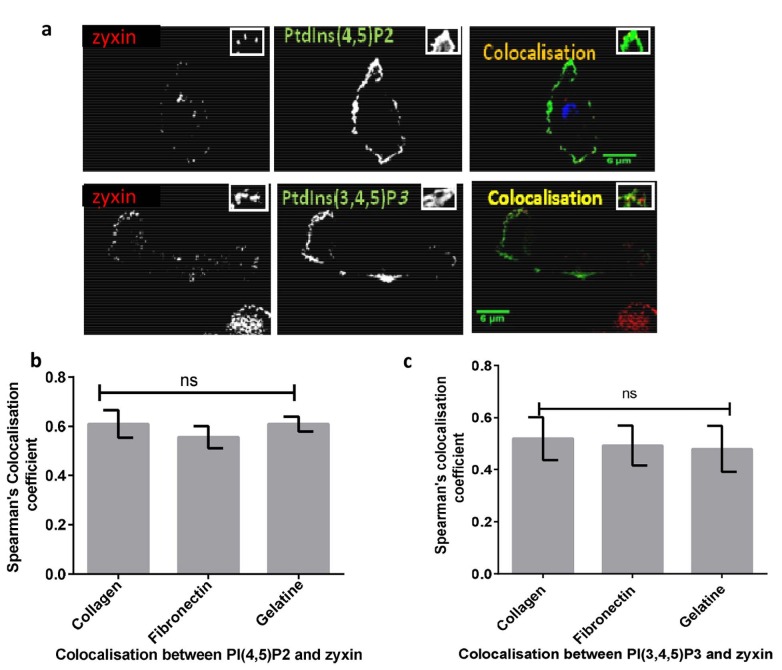
(a) Spatial colocalisation between PtdIns(4,5)P2\ PtdIns(3,4,5)P3 and a single FA in MDA-MB-231 cells cultured on different surfaces. MDA-MB-231 cells plated on 20-μg/mL fibronectin, 0.2% w/v gelatine and 2-mg/mL collagen were cotransfected with PLC𝛅1-PHGFP\ Btk-PH-GFP (green) and zyxin-mCherry(red). The highlighted areas show a magnification of the spatial colocalisation region between PtdIns(4,5)P2 and a single FA. Three independent experiments were performed. Representative pictures are shown. The scale bar is indicative of a 6-μm length. Quantification of spatial colocalisation between (b) PtdIns(4,5)P2, (c) PtdIns(3,4,5)P3, and zyxin. Spearman’s (rho) correlation coefficient analysis was used to determine the strength colocalisation between PtdIns(4,5)P2 and zyxin. The pooled data shows a colocalisation between PtdIns(4,5)P2 and zyxin, as well as between PtdIns(3,4,5)P3 and zyxin on different surfaces.

The distributions of PtdIns(4,5)P2 and PtdIns(3,4,5)P3 were monitored in MDA-MB-231 cells with the aid of PLC;;1- PH-GFP/mCherry and Btk-PH-GFP/mCherry biosensors, respectively. To evaluate the potential impact of different ECM compositions on PtdIns(4,5)P2 and PtdIns(3,4,5)P3 in relation to zyxin, MDA-MB-231 cells cultured on different ECM surfaces were assessed for potential spatial redistribution of PtdIns(4,5)P2\PtdIns(3,4,5)P3 with regards to zyxin (Figure 3a). Colocalisation analyses of the distribution of PtdIns(4,5)P2 and zyxin gave rise to a Spearman’s coefficients of 0.61 ± 0.05, 0.55 ± 0.04, and 0.61 ± 0.03 in cells grown on collagen, fibronectin, and gelatine, respectively (Figure 3b). The coefficients resulting from the spatial distributions of PtdIns(3,4,5)P3 and zyxin in cells cultured in the presence of collagen, fibronectin, and gelatine were 0.52 ± 0.08, 0.49 ± 0.07, and 0.48 ± 0.08, respectively (Figure 3c).

### 3.2. Changes of local PtdIns(4,5)P2 and PtdIns(3,4,5)P3 levels during FA assembly and disassembly

**Figure 4:**
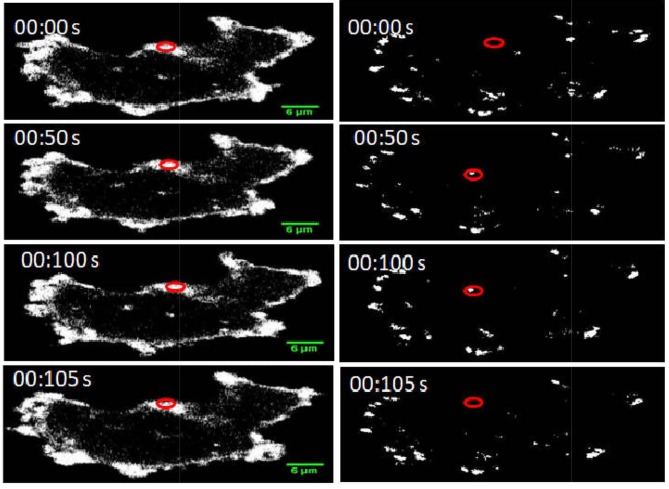
Quantification of the local levels of PtdIns(4,5)P2 within a single FA. Confocal live imaging showing the local levels of PtdIns(4,5)P2 throughout the turnover of zyxin turnover. MDA-MB-231 cells were plated on collagen (2mg/mL) and cotransfected with PLC𝛅1-PH-GFP and zyxin-RFP. Z-Stacks were taken at a 0.15-μm distance with time-lapse series acquired over a 10-min period with an interval of 15 s. The first column refers to local levels of PtdIns(4,5)P2, and the second column refers to zyxin assembly and disassembly. The highlighted areas show the ROI that was drawn closely around the zyxin and the PtdIns(4,5)P2 to measure the specific localisation of local levels of PtdIns(4,5)P2 within a single zyxin. The zyxin selected for measuring should be absent both at the beginning and end of the time-lapse series.

Since the use of various ECM gave rise to identical spatial distribution across PtdIns(4,5)P2 and PtdIns(3,4,5)P3 in relation to zyxin, further experiments investigating temporal changes of PtdIns(4,5)P2 and PtdIns(3,4,5)P3 levels throughout the lifetime of FA were undertaken with MDA-MB-231 cells cultured on a collagen surface. Cells transfected with either PLC;;1-PH-GFP/PLC;;1-PH-mCherry and RFP-zyxin/RFP-paxillin or Btk-PH-GFP/Btk-PH-mCherry and RFP-zyxin/RFP-paxillin were visualised 24 h after transfection using a confocal microscope for live cell imaging. GFP and RFP (or mCherry) were detected, after excitation/emission at 488 nm/510 nm and 568 nm/590 nm, respectively. Z-Stacks were taken at a 0.15-μm distance, and time-lapse series were acquired over a 10 min period with an interval of 15 s in order to accurately determine the colocalisation between PtdIns(4,5)P2 or PtdIns(3,4,5)P3 and zyxin and between PtdIns(4,5)P2 or PtdIns(3,4,5)P3 and paxillin. The local levels of PtdIns(4,5)P2 and PtdIns(3,4,5)P3 were measured at different time points and at different focal planes within and around a single FA during assembly and disassembly. Quantitative analysis of these images obtained from live cells was performed using Image J. In the zyxin/paxillin channel, an ROI was selected in the surrounding vicinity of individual zyxin/paxillin. The same selection was applied to the GFP or mCherry channel of PtdIns(4,5)P2 and PtdIns(3,4,5)P3 to determine their specific localisation within a single FA during its turnover. The intensities of fluorescence of both PtdIns(4,5)P2 and PtdIns(3,4,5)P3 were measured within the ROI of zyxin/paxillin throughout the course of the complete lifetime (turnover cycle) of the zyxin/paxillin (Figure 4).

**Figure 5:**
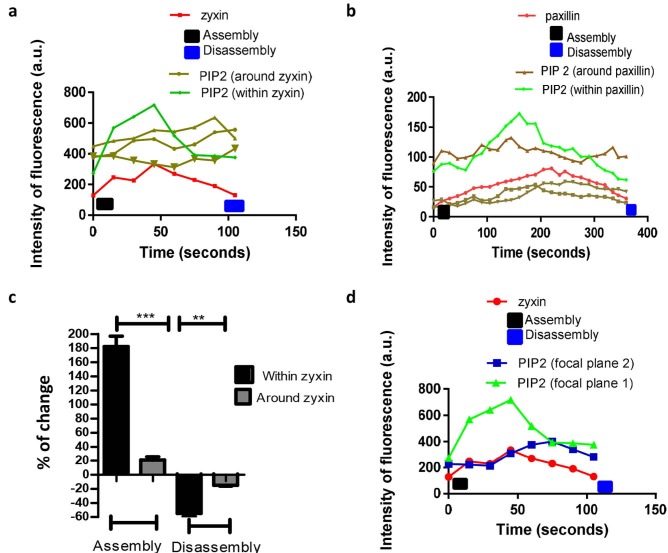
Dynamic change of local levels of PtdIns(4,5)P2 within and around FA turnover. The green curve refers to the level of PtdIns(4,5)P2 within FAs, The brown curves refer to the level of PtdIns(4,5)P2 around zyxin and paxillin turnover, and the red curve refers to zyxin turnover over time (105 s). (a/b) Dynamic change of PtdIns(4,5)P2 within and around zyxin and paxilin during assembly and disassembly. The local levels of PtdIns(4,5)P2 were increased within zyxin and paxillin during assembly and declined during disassembly, while around zyxin the local levels of PtdIns(4,5)P2 were slightly changed. (c) Percentage of change of local levels of PtdIns(4,5)P2 within zyxin was significantly higher than around zyxin. (c) Quantification of the dynamic change of PtdIns(4,5)P2 at different focal planes within zyxin during assembly and disassembly. Data are representative of N = 30 FA in 10 migrating MDA-MB-231 cells. Statistical analysis was performed with the aid of one-way ANOVA with Tukey’s multiple comparison test. P-values showing statistical difference were labelled as (**) for P < 0.01 and (***) for P < 0.0005. Data presented as mean ± SEM of 3 independent experiments.

Overall, PtdIns(4,5)P2 colocalised with zyxin or paxillin was found to increase and decline in synchronicity with assembly and disassembly of zyxin or paxillin. PtdIns(4,5)P2 around zyxin/paxillin showed no variation during the assembly and disassembly of zyxin/paxillin (Figures 5a and 5b). Next, we measured the percentage of change of PtdIns(4,5)P2 within and around zyxin and paxillin; the local levels of PtdIns(4,5)P2 within zyxin were significantly higher compared to that of PtdIns(4,5)P2 in the surrounding of zyxin as the percentage of change of PtdIns(4,5)P2 within zyxin was found to be 182.0 ± 14.8% and –55.0 ± 4.5% during FA assembly and disassembly, respectively, while PtdIns(4,5)P2 around zyxin was found to be 21.0 ± 4.7% and –15.0% ± 6.7 during FA assembly and disassembly, respectively (Figure 5c). The local levels of PtdIns(4,5)P2 was measured at different focal planes were found to increase during zyxin assembly and decline during the FA disassembly (Figure 5d).

**Figure 6:**
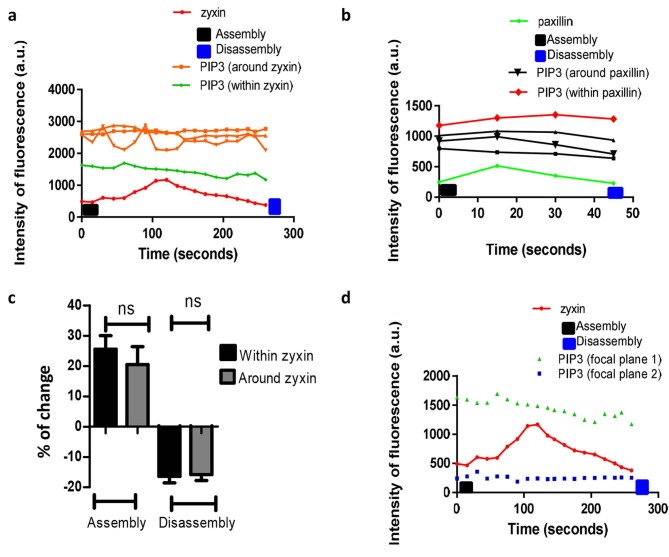
Dynamic change of local levels of PtdIns(3,4,5)P3 within and around FA turnover. The green curve refers to the level of PtdIns(3,4,5)P3 within FA, the brown curves refer to the level of PtdIns(3,4,5)P3 around zyxin and paxillin turnover, and the red curve refers zyxin and paxillin turnover over time (105 s). (a/b) Quantification of the dynamic change of PtdIns(3,4,5)P3 within and around zyxin and paxillin assembly and disassembly. PtdIns(3,4,5)P3 was at a constant level during zyxin and paxillin turnover. (c) Percentage change of local levels of PtdIns(3,4,5)P3 within and around zyxin was not significantly different. (d) Dynamic change of PtdIns(3,4,5)P3 within zyxin and paxillin at different focal plane. Data are representative of N = 30 FA intensity profiles in 10 migrating MDA-MB-231 cells. Statistical analysis was performed using a one-way ANOVA with Tukey’s multiple comparison test. Statistical significance was accepted at P < 0.05. Data presented as mean ± SEM of 3 independent experiments.

The dynamic changes of local levels of PtdIns(3,4,5)P3 within and around zyxin/paxillin were found to be constant throughout, the assembly and disassembly of zyxin/paxillin (Figures 6a and 6b). The evaluation of the percentage of change of local levels of PtdIns(3,4,5)P3 within and around zyxin showed that the percentage of change of PtdIns(3,4,5)P3 within zyxin was 26.0 ± 4.5% and –16.0 ± 2.2% during its assembly and disassembly, while PtdIns(3,4,5)P3 around zyxin gave rise to percentage of changes of 20.0 ± 5.9% and –15.8 ± 1.9% (Figure 6c). The local levels of PtdIns(3,4,5)P3 measured at different focal plane of the plasma membrane were found to be constant during both the assembly and disassembly of zyxin (Figure 6d).

## 4. Discussion

In this study, we investigated the spatiotemporal distribution of PtdIns(4,5)P2 and PtdIns(3,4,5)P3 during the assembly and disassembly of FAs. The direct visualisation of plasma membrane inositol lipids has proven to be challenging when using immunological labelling. Given that PH domains are known to bind to different phosphoinositides, when labelled fluorescently, these PH-domains can serve as a powerful tool to detect and visualise inositol lipids by confocal microscopy. Although some PH domains may possess poor affinity for inositol lipids, in the present study we used PLCδ- PH-GFP/mCherry and Btk-PH-GFP/mCherry since both biosensors bind to PtdIns(4,5)P2 and PtdIns(3,4,5)P3, respectively, with high affinity and selectivity (Vrnai & Balla, 1998, Varnai et al., 1999, Manna et al., 2007, Balla and Varnai, 2009).

To investigate the spatial distribution and temporal changes of inositol lipids during FAs assembly and disassembly, both biosensors were used to monitor local changes of PtdIns(4,5)P2 and PtdIns(3,4,5)P3, as well as their localisation in plasma membrane. Our result showed that both PLCδ-PH-GFP/mCherry and Btk-PH-GFP/ mCherry are mostly recruited to the plasma membrane (Figure 3a) of MDA-MB-231 cells (Vrnai and Balla, 1998; Stauffer et al., 1998; Vrnai et al., 1999; Matsuda et al., 2001; Manna et al., 2007).

Furthermore, our data showed that distributions of PtdIns(4,5)P2 and PtdIns(3,4,5)P3 were moderately correlated with FA proteins such as zyxin and paxillin in cells grown upon different surfaces (Figures 3b and 3c). This spatial colocalisation may suggest that PtdIns(4,5) P2 and PtdIns(3,4,5)P3 could be associated with FAs dynamics through the recruitment of FA proteins which could strengthen the interaction between FAs and integrins (Gilmore & Burridge, 1996; Le et al., 2015). Previous studies have shown that the initiation of the formation of FAs depends on the interaction between integrins and ECM. Distinct isoforms of integrins recognise different types ECM (Hynes, 2002). For example, α5β1, αvβ1, and α4β1, recognise fibronectin, and α1β1 and α2β1 recognise collagen. Therefore, it is plausible that a different ECM may exert different effects on FA dynamics and cell motility (Huttenlocher & Horwitz, 2011). Here we investigated the effect of different extracellular surfaces (upon which cells cultured) on the spatial distribution of FAs, PtdIns(4,5) P2 and PtdIns(3,4,5)P3. Our results showed that the spatial distribution of PtdIns(4,5)P2 and PtdIns(3,4,5)P3 correlate with zyxin in the same manner across various ECM surfaces. Thus, this suggests that different types of ECM do not affect the colocalisation between PtdIns(4,5) P2 and FAs or PtdIns(3,4,5)P3 and FAs in MDA-MB-231 cells.

Our results also showed that the local levels of PtdIns(4,5)P2 within FAs increased with FA proteins assembly and declined with FA proteins disassembly (Figure 4). As previously demonstrated, talin is responsible in recruiting PIP5K into the plasma membrane during FA assembly. Once activated, PIP5Kγ (the main enzyme generating PtdIns(4,5)P2 in the plasma membrane) phosphorylates phosphatidylinositol 4-phosphate (PtdIns4P) to produce PtdIns(4,5)P2 (Legate et al., 2011; Zhaofei Wu et al., 2011; Izard & Brown, 2016). During the early stages of FA assembly, the local increase of PtdIns(4,5)P2 mediated by PIP5Kγ leads to the activation of talin, following PtdIns(4,5)P2 binding. This causes talin to be recruited onto FA sites, which in turn allows forvinculin activation following its interaction with the activated talin (Izard & Brown, 2016; Yuan et al., 2017). At later stages of FA assembly, other proteins such as vasodilator-stimulated phosphoprotein can also be recruited by vinculin (Garvalov et al., 2003). Conversely, the accumulation of PtdIns(4,5)P2 pool at later stages of FA assembly may contribute to FA disassembly (Izard & Brown, 2016; Yuan et al., 2017). PtdIns(4,5)P2 has been reported to potentially strengthen the interaction between FAs and integrin during the assembly phase (Saltel et al., 2009). The decline of local levels of PtdIns(4,5)P2 with FA disassembly could be due to the loss of PIP5Kγ activity during the detachment of FAs from the ECM since talin is deactivated during FA disassembly, thus causing PIP5Kγ deactivation (Legate et al., 2011; Zhaofei Wu et al., 2011; Izard & Brown, 2016). The accumulated PtdIns(4,5) P2 in the FA sites may result in the recruitment of both PI3K and PLC, where PI3K produces PtdIns(3,4,5)P3 through the phosphorylation of PtdIns(4,5)P2, while PLC converts PtdIns(4,5)P2 to DAG and IP3(Gericke et al., 2013), thereby resulting in the reduction of local levels of PtdIns(4,5)P2 during FA disassembly (Izard & Brown, 2016). Taken together, it is most likely that PtdIns(4,5)P2 may be implicated in the regulation of FA assembly and its disassembly (Chinthalapudi et al., 2014).

The local levels of PtdIns(4,5)P2 in the immediate surrounding of FA proteins was found to remain constant throughout the assembly and disassembly of FAs (Figure 5a). The percentage change of local levels of PtdIns(4,5)P2 within FAs was significantly higher than the PtdIns(4,5) P2 around FAs (Figures 5c and 5d), suggesting that an enrichment of PtdIns(4,5)P2 is restricted within specific regions. This is consistent with other studies showing that the enrichment of PtdIns(4,5)P2 varies between regions which contain signalling kinases requiring PtdIns(4,5)P2 to perform their function (Wang & Richards, 2012; C. Ji et al., 2015).

Regarding local levels of PtdIns(3,4,5)P3 within and around FAs, our results showed that PtdIns(3,4,5) P3 remained constant throughout the assembly and the disassembly of FA (Figure 6). This may indicate a potential limit to the sensitivity of the currently used method to detect PtdIns(3,4,5)P3. The abundance of PtdIns(3,4,5)P3 is 95%98% less than that of PtdIns(4,5)P2 (500020,000 molecules/m2) in the plasma membrane (Falkenburger et al., 2010; Balla, 2013).Given such low levels, PtdIns(3,4,5) P3 may be undetectable by the biosensors. Therefore, a dynamic change of local levels of PtdIns(3,4,5)P3 during FA assembly and disassembly may go unnoticed. This interpretation is in agreement with the work of Zhaofei Wu et al., who showed that local levels of PtdIns(4,5)P2 decline to ~40% as a result of PIPKIγsh RNA knockdown, whilst local levels of PtdIns(3,4,5)P3 could not be detected (Zhaofei Wu et al., 2011). PtdIns(4,5)P2, but not PtdIns(3,4,5)P3, has also been shown to be essential for FA turnover (Zhaofei Wu et al., 2011) as both differ in their temporal changes and spatial distribution (Insall & Weiner., 2001). PtdIns(3,4,5)P3 might play an indirect role in cell migration through the activation of other signalling pathways (Insall & Weiner., 2001), although its function may not be fundamental in FA turnover (Qin et al., 2009).

**Figure 7:**
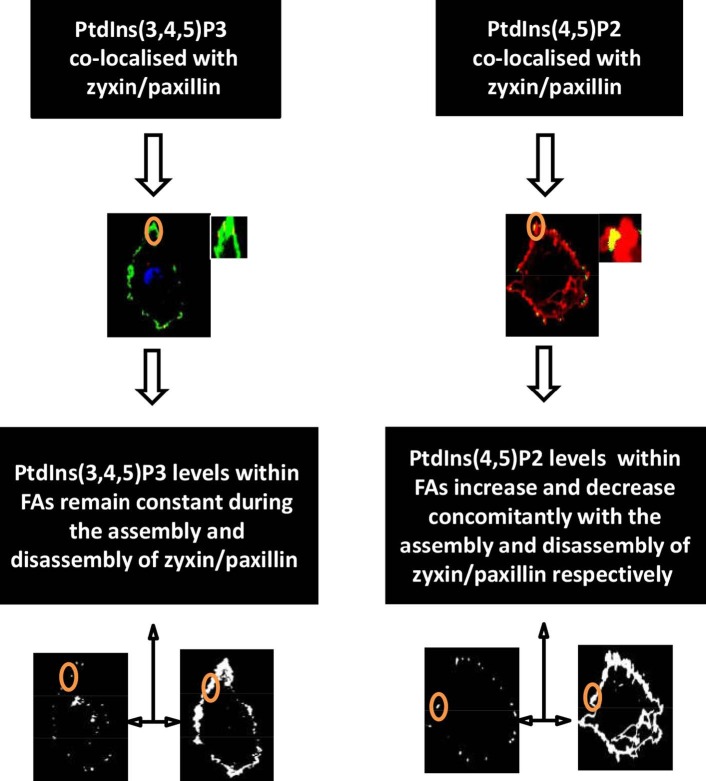
Diagram summarising the main findings of this study. Both PtdIns(4,5)P2 and PtdIns(3,4,5)P3 were found to be colocalised with zyxin and paxillin; however, only the level of PtdIns(4,5)P2 was found to change with the assembly and disassembly of FA proteins.

The rational for measuring PtdIns(4,5)P2 and PtdIns(3,4,5)P3 levels within FAs at different focal planes was to determine an accurate colocalisation with different FA proteins across the plasma membrane. FAs are located on the cytosolic side of the plasma membrane and constitute a 60-nm thickness (Kanchanawong et al., 2010). Given that paxillin is nearer to the plasma membrane compared to zyxin, Z-stacks were performed on live cells to determine the localisations of PtdIns(4,5) P2, PtdIns(3,4,5)P3, and those FA proteins across different focal planes of the plasma membrane. Our data showed that the changes of the local levels of PtdIns(4,5)P2 and PtdIns(3,4,5)P3 during the assembly and disassembly of FA proteins were overall comparable in both focal planes. A graphical figure summarizing the findings in this paper can be found in Figure 7.

Furthermore, similar results were found with vinculin and talin (data not shown). Zyxin-RFP/GFP and paxillin- RFP/GFP were presented here because they were found to be representative of FA proteins in our experimental design.

This study could have benefited from the use of a control condition. For example, the use of a binding- deficient version of the probe, such as PLCδ1-GFP with the mutant PH domain (R40L) could have been used as the negative control for the PLCδ1-PH domain (wild-type) (Várnai & Balla, 1998). R40L mutation in the wild-type δ1-PH domain prevents the cytosol translocation of PLC. In addition, other PH domain mutants reported by Vrnai and Balla that are unable to bind with phosphoinositides (Várnai & Balla, 1998) could have been used to ensure that any observed changes in the PtdIns(4,5)P2 level is not due to morphological changes or the size of the membrane within and around a single FA turnover (Tamas Balla & (Várnai, 2009). Although R40L mutation prevents PtdIns(4,5)P2 from binding to the PH domain, but does not affect its interaction with other plasma membrane proteins, the use of membrane protein markers such as the myristoylated/palmitoylated GFP (PM-GFP) or GFP- CAAX, could have served to demonstrate that PLCδ-PH- GFP does not bind to membrane proteins in general but rather reflects direct changes within FA (Madugula & Lu, 2016).
